# Escitalopram alters tryptophan metabolism, plasma lipopolysaccharide, and the inferred functional potential of the gut microbiome in deer mice showing compulsive-like rigidity

**DOI:** 10.1017/neu.2025.16

**Published:** 2025-04-03

**Authors:** Larissa Karsten, Brian H. Harvey, Dan J. Stein, Benjamín Valderrama, Thomaz F.S. Bastiaanssen, Gerard Clarke, John F. Cryan, Rencia van der Sluis, Heather Jaspan, Anna-Ursula Happel, De Wet Wolmarans

**Affiliations:** 1 Centre of Excellence for Pharmaceutical Sciences, Faculty of Health Sciences, North-West University, Potchefstroom, South Africa; 2 SAMRC Unit on Risk and Resilience in Mental Disorders, Department of Psychiatry and Mental Health and Neuroscience Institute, University of Cape Town, Cape Town, South Africa; 3 IMPACT Strategic Research Centre, School of Medicine, Barwon Health, Deakin University, Geelong, Australia; 4 APC Microbiome Ireland, University College Cork, Cork, Ireland; 5 Department of Anatomy and Neuroscience, University College Cork, Cork, Ireland; 6 Department of Psychiatry and Neurobehavioural Science, University College Cork, Cork, Ireland; 7 Biomedical and Molecular Metabolism Research (BioMMet), North-West University, Potchefstroom, South Africa; 8 Department of Pathology, University of Cape Town, Cape Town, South Africa; 9 Institute of Infectious Disease and Molecular Medicine (IDM), University of Cape Town, Cape Town, South Africa; 10 Seattle Children’s Research Institute, Seattle, WA, USA; 11 Departments of Pediatrics and Global Health, University of Washington, Seattle, WA, USA

**Keywords:** Deer mouse, obsessive-compulsive disorder, rigidity, serotonin, microbiome

## Abstract

**Objective:**

Compulsive-like rigidity may be associated with hyposerotonergia and increased kynurenine (KYN) pathway activity. Conversion of tryptophan (TRP) to KYN, which may contribute to hyposerotonergia, is bolstered by inflammation and could be related to altered gut microbiota composition. Here, we studied these mechanisms in a naturalistic animal model of compulsive-like behavioural rigidity, that is, large nest building (LNB) in deer mice (*Peromyscus* sp.).

**Methods:**

Twenty-four (24) normal nest building (NNB) and 24 LNB mice (both sexes) were chronically administered either escitalopram (a selective serotonin reuptake inhibitor; 50 mg/kg/day) or a control solution, with nesting behaviour analysed before and after intervention. After endpoint euthanising, frontal cortices and striata were analysed for TRP and its metabolites, plasma for microbiota-derived lipopolysaccharide (LPS) and its binding protein (lipopolysaccharide binding protein), and stool samples for microbial DNA.

**Results:**

LNB, but not NNB, decreased after escitalopram exposure. At baseline, LNB was associated with reduced frontal cortical TRP concentrations and hyposerotonergia that was unrelated to altered KYN pathway activity. In LNB mice, escitalopram significantly increased frontal-cortical and striatal TRP without altering serotonin concentrations. Treated LNB, compared to untreated LNB and treated NNB mice, had significantly reduced plasma LPS as well as a microbiome showing a decreased inferred potential to synthesise short-chain fatty acids and degrade TRP.

**Conclusions:**

These findings support the role of altered serotonergic mechanisms, inflammatory processes, and gut microbiome involvement in compulsive-like behavioural rigidity. Our results also highlight the importance of gut-brain crosstalk mechanisms at the level of TRP metabolism in the spontaneous development of such behaviour.

## Graphical abstract

**Figure:**
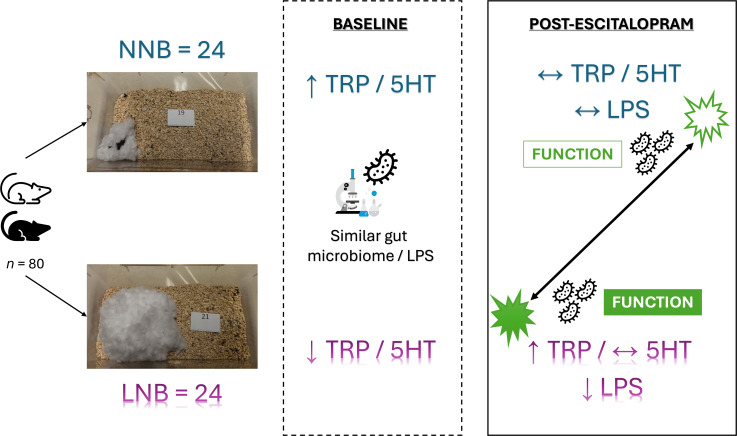

## Significant outcomes

- Behavioural rigidity is associated with decreased brain tryptophan, but not kynurenine metabolites.
- Escitalopram increases brain tryptophan, but not serotonin, in behaviourally rigid mice, highlighting a non-serotonergic impact of the gut microbiota on rigidity.
- Rigidity predicts changes in plasma lipopolysaccharide (LPS) and gut microbiome function after escitalopram treatment, but is not directly founded upon altered LPS, that is, gut-microbiota-related inflammation, at baseline.

## Limitations

- Since mice showing phenotype-specific variation in nesting scores were included in the large nest building (LNB) and NNB cohorts, larger groups will allow the study of sex-dependent effects and thereby enable correlational analysis of nesting behaviour and its association with different biological parameters.
- A post-drug-exposure lipopolysaccharide (LPS) challenge in escitalopram-exposed NNB and LNB mice would have been valuable to trace a direct relationship between LPS and nesting expression. Similarly, an investigation of inflammatory processes, that is, IDO and TDO activity, cytokine expression, and cortisol release would have been beneficial.
- The microbiota of *P. maniculatus bairdii* is not a well-characterised ecosystem yet. It is therefore likely underrepresented in the databases used for taxonomic identification and inferred functional potential.

## Highlights

- Behavioural rigidity is associated with decreased brain tryptophan, but not kynurenine metabolites.

- Rigidity uniquely predicts changes in plasma lipopolysaccharide and gut microbiome function after escitalopram treatment.

- In rigid mice, escitalopram increases brain tryptophan, but not serotonin, highlighting a non-serotonergic impact of gut microbiota on rigidity.

## Introduction

Behavioural rigidity, as found in obsessive-compulsive disorder (OCD) (Ramakrishnan *et al*., 2022) and autism spectrum disorder (ASD) (Poljac *et al*., 2017), is variably associated with aberrant cortico-striatal serotonergic (Luo *et al*., 2024) and glutamatergic (Naaijen *et al*., 2017) signalling. Serotonin is derived from the essential amino acid, tryptophan (TRP) (Lissemore *et al*. (2018)), and is synthesised *de novo* in the central nervous system from dietary sources. TRP is metabolised along distinct mammalian pathways. The kynurenine (KYN) pathway, through which TRP is converted to KYN and its downstream metabolites, accounts for more than 90% of TRP breakdown. In contrast, TRP can be converted to serotonin via the actions of TRP hydroxylase 1 and 2 (Höglund *et al*., 2019). This process accounts for only 1–3% of TRP breakdown. Thus, increased KYN pathway activity may reduce the available TRP needed for the synthesis of serotonin (Sun *et al*., 2020).

Along the KYN pathway, TRP is first converted to KYN through the actions of tryptophan-2,3-dioxygenase (TDO) and indoleamine-2,3-dioxygenase (IDO) in hepatic and extrahepatic tissue, respectively (Stone *et al*., 2012; Gao *et al*., 2018). KYN is subsequently metabolised to anthranilic acid, 3-hydroxykynurenine (OHK), and kynurenic acid (KYNA). OHK is then degraded to either quinolinic acid (QA) or picolinic acid (Lovelace *et al*., 2017). Although several KYN metabolites display neuroactive properties, KYNA and QA are the two primary neuroactive metabolites, acting as glutamate *N*-methyl-D-aspartate (NMDA) receptor antagonists and agonists, respectively (Schwarcz and Köhler, 1983; Schwarcz *et al*., 2012).

The expression and activity of IDO and TDO are accelerated under inflammatory and stress states, respectively (Strasser *et al*., 2017). An important trigger of inflammation is lipopolysaccharide (LPS), an endotoxin derived from the cell walls of gram-negative bacteria that activates the innate immune system via its interaction with toll-like receptor 4 (TLR4) (Rosadini and Kagan, 2017). Increased plasma LPS concentrations (Fujigaki *et al*., 2001), which are associated with systemic infection, gut microbiota composition alterations, and increased gut permeability (Hasegawa *et al*., 2015), are therefore an important driver of bolstered IDO activity. Closely related to altered LPS concentrations are dynamic shifts in the expression of lipopolysaccharide binding protein (LBP), an endogenous acute phase polypeptide that is synthesised in response to increased concentrations of inflammatory cytokines and other inflammatory components, including LPS (Zweigner *et al*., 2006). The main function of LBP, after binding to LPS, is to present LPS to other cellular and humoral components of the immune system, thereby bolstering the immune response (Zweigner *et al*., 2006). Several central nervous system disorders are associated with gut microbiota-related changes in plasma LPS and LBP concentrations that variably present in combination with altered KYN pathway activity. These include Parkinson’s disease (Hasegawa *et al*., 2015), ASD, and anxiety disorders (Just *et al*., 2021).

A handful of exploratory studies in children and adults presenting with conditions of behavioural rigidity, for example, OCD (Heyes *et al*., 1992), tic disorder (Hoekstra *et al*., 2007), Tourette’s syndrome (Rickards *et al*., 1996), and ASD (Carpita *et al*., 2023), have not reported any differences in KYN pathway activity or its metabolites. That said, clinical data pertaining to TRP metabolism in these conditions remain scant. Although theories of the potential involvement of altered KYN pathway dynamics in OCD have been based on findings of immune abnormalities (Rotge *et al*., 2010; Teixeira *et al*., 2014; Kant *et al*., 2018; Marazziti *et al*., 2018b; Cosco *et al*., 2019), there is no direct evidence of inflammation-mediated neurochemical alterations in OCD, or of changes in gut microbiome composition that could lead to alterations in plasma LPS and LBP.

The North American deer mouse *(Peromyscus maniculatus)* is commonly used to investigate the potential mechanisms underlying immunological resilience and infection tolerance (Milovic *et al*., 2024) and those underpinning contributions of the gut microbiome to overall health (Zucker, 2023; Mistrick *et al*., 2024). Also, subpopulations of laboratory housed prairie deer mice *(P. maniculatus bairdii)* spontaneously develop phenotypically distinct persistent and repetitive behaviours, that is, high motor stereotypy (40–45% of mice; Hadley *et al*. (2006); Burke *et al*. (2022); Davis *et al*. (2023)), large nesting behaviour (LNB) (30–35% of mice; Stoppel *et al*. (2024)), and high marble burying behaviour (10–15% of mice de Brouwer *et al*. (2020a)). These phenotypes are displayed by mice of both sexes and are identified by means of bidirectional separation between mice showing these behaviours and those that do not. Moreover, deer mice have been studied for their resemblance to compulsive-like rigidity (for a detailed review of the model’s relevant validity, please refer to Scheepers *et al*. (2018); Theron *et al*. (2024)). For this purpose, bidirectional separation is useful. The application of ‘repetition’ and between-test ‘persistence’ to categorise mice into ‘normal’ and ‘rigid’ cohorts (Supplementary Fig. S1; collated nesting data of 942 mice studied in our lab) delivers a repeatable framework for behavioural separation that can be exploited to investigate psychobiological uniqueness in mice displaying rigid behaviours. For example, LNB mice identified in this manner previously showed a distinct gut microbiota composition that is proposed to associate with an immune-inflammatory profile, compared to normal nesting (NNB) mice (Scheepers *et al*., 2019). Further, LNB parallels central serotonergic, dopaminergic, and adenosinergic perturbations (de Brouwer *et al*., 2020a; Saaiman *et al*., 2023), with LNB, but not normal nest building (NNB) behaviour showing moderation after chronic, high-dose oral exposure to the selective serotonin reuptake inhibitor (SSRI), escitalopram (De Brouwer *et al*., 2020b). At the level of cognition, LNB mice show impaired cognitive flexibility (Hurter *et al*., 2023; Marx *et al*., 2024) and decreased risk aversion (de Brouwer *et al*., 2020c; Wolmarans *et al*., 2022), speaking to dysfunctional executive decision making that could contribute to rigidity (Servaas *et al*., 2021; Ramakrishnan *et al*., 2022). However, to what extent alterations in the gut microbiome of LNB mice may be associated with changes in brain serotonin levels, TRP metabolism, and plasma LPS and LBP is unknown.

Therefore, we aimed to build on earlier findings by investigating whether LNB and NNB deer mice present with distinct cortico-striatal TRP-related metabolic profiles. Further, based on the established relationship between systemic inflammation and TRP breakdown, we also aimed to investigate how such differences might be associated with changes in the microbiota composition and plasma LPS and LBP concentrations. Last, we sought to explore the biobehavioural actions of escitalopram in the model by investigating its potential effects on central TRP metabolism, gut microbiota composition, and plasma LPS and LBP.

## Materials and methods

### Mice

Considering that approximately 30% of deer mice *(Peromyscus maniculatus bairdii)* engage in LNB behaviour (de Brouwer *et al*., 2020a; Wolmarans *et al*., 2022), 80 mice of both sexes (first and second generation), aged 12–14 weeks at the onset of experimentation, were randomly selected from the offspring of 20 breeding pairs (of which the nesting phenotype were unknown at the time of pairing) and screened for nesting behaviour. Mice were bred and housed at the vivarium (SAVC reg: FR15/13458; AAALAC accreditation file: 1717) of the North-West University (NWU), Potchefstroom, South Africa. All experiments were conducted in said facility. Ethical approval for this work was obtained from the *AnimCare* Research Ethics Committee of the NWU (**NWU-00523-20-A5**). Deer mice were housed in individually ventilated cages [35 (l) × 20 (w) × 13 (h) cm; Techniplast® S.P.A., Varese, Italy] that were maintained at 23°C on a normal 12-h light/dark cycle (lights on at 06:00). Cages were cleaned, and new corncob bedding provided, weekly. Throughout the course of the study, food and water (or drug solutions) were available ad lib. All experimental procedures were conducted in accordance with the guidelines of the South African National Standard (SANS) for the Care and Use of Animals for Scientific Purposes (SANS 10386).

### Nesting assessment

Before any other intervention was made, all 80 mice were screened for nesting behaviour (baseline expression) over a 7-day period, since nesting activity varies between consecutive days. From these, the respective NNB and LNB mice were selected for further study (see later). From this point onwards, mice were single-housed through study termination (De Brouwer *et al*., 2020b). Each day, an excess of pre-weighed, unscented cosmetic cotton wool was introduced to the roof of each housing cage between 07:00 and 08:00. Since mice mostly engage in nesting behaviour during the few hours before dawn (or lights on) (Jirkof, 2014), built nests were also only removed and discarded between 07:00 and 08:00. Thus, mice had access to the nesting material for at least 23 h of each day. Every day, the unused cotton wool in the roof of the cage was weighed to calculate the daily usage. These seven daily values (in grams) were added, and a total nesting score was calculated for each mouse (Wolmarans *et al*., 2016). Mice included in the LNB cohort were those that expressed nesting behaviour of which the total nesting scores broadly clustered above the upper 75^th^ percentile of the average total nesting score distribution and that also showed the lowest degree of between-day variance (as reflected by the percentage coefficient of variance; % CV; Supplementary Fig. S2A; *n* = 24; equally distributed between sexes). Conversely, mice selected for NNB behaviour (*n* = 24; as far as possible also equally distributed between sexes) were those with nesting scores that clustered between the 25^th^ and 50^th^ percentile of the total nesting score distribution (Supplementary Fig. S2A). These group sizes were based on extensive prior study in our laboratory that investigated NNB and LNB as distinguished by means of bidirectional separation (Wolmarans *et al*., 2022; Hurter *et al*., 2023; Marx *et al*., 2024). To ensure that bidirectional separation between cohorts is adequately established, the remaining 32 mice were either used for unrelated studies or euthanised, as described before (Marx *et al*., 2024). During periods of nest building analysis, mice were not provided with any additional form of nesting material. Nesting assessment was again assessed after 28 days of continued water or escitalopram exposure, over another 7 days of control or drug exposure.

### Drug administration

The selected 24 NNB and 24 LNB mice of both sexes were randomly assigned to two different exposure groups (*n* = 12 per cohort per exposure group). One group of each cohort was exposed to normal tap water (control) while the other group was exposed to high-dose escitalopram (50 mg/kg/day) for 35 days, as described before (Wolmarans *et al*., 2013; de Brouwer *et al*., 2020a; Wolmarans *et al*., 2022; de Ridder *et al*., 2022; Burke *et al*., 2022). Escitalopram oxalate (BLD Pharm®, Shanghai, China) was administered in the drinking water at a concentration of 25.4 mg/100 ml, calculated according to the average daily water intake of deer mice (0.25 ml/g/day; de Brouwer *et al*. (2020a); de Brouwer *et al*. (2020c); Wolmarans *et al*. (2022)) to deliver the desired 50 mg/kg/day dose. Fresh drug solutions were constituted daily, and the fluid intake of both drug- and control-exposed mice was measured to confirm drug intake. Oral drug administration via the drinking water is the preferred administration route in deer mice since intraperitoneal injections or oral gavage over a chronic period is detrimental to health (Wolmarans *et al*., 2013). Mice were exposed to these interventions for at least 28 days before the post-exposure nesting assessment commenced.

### Sample collection

Between 06:00 and 07:00 on the morning following the last night of post-exposure nesting assessment, fresh stool samples were collected using sterile tweezers and snap frozen in liquid nitrogen. Mice were then euthanised by means of cervical dislocation (Underwood and Anthony, 2020). Whole blood was collected in ethylenediamine tetra-acetic acid-containing vacutainers (Becton, Dickinson and Company®, Sandton, South Africa), the colon and brains were removed on ice, the frontal cortices and striata were dissected, and samples were snap frozen in liquid nitrogen. Blood samples were centrifuged at 1000 RCF for 10 min at 4°C, and the plasma was collected. All stool, plasma, and brain samples were stored at −80°C until the day of analysis.

### Analysis of TRP and its metabolites

#### Reagents, chemicals, and instrumentation

L-TRP, L-KYN, KYNA, QA, serotonin (as creatinine sulphate), 5-hydroxyindoleacetic acid (5-HIAA), ethyl-4-hydroxy-2-quinolinecarboxylate (EHQC; as internal standard), and LC/MS grade methanol, formic acid, acetonitrile (CAN), and glacial acetic acid were all purchased from Merck® (Johannesburg, South Africa).

#### Sample preparation and analysis

A stock solution of the internal standard was prepared at a concentration of 100 µg/mL using a solvent mixture of 0.1 M formic acid in ACN, after which a working internal standard solution with a final concentration of 250 ng/mL was prepared from the stock solution using the same solvent mixture. The working solution was also used for the preparation of the different biological sample matrices. Brain and colon samples were individually weighed prior to preparation. 200 µL of the internal standard was added to each of the samples, followed by homogenisation (two rounds of sonication for 12 s, at an amplitude of 14 µ; MSE® ultrasonic disintegrator, Nuaillé, France). Mixtures were left on ice for 20 min to complete protein precipitation and centrifuged at 20 817 RCF for 20 min at 4°C. A Kinetix C18 analytical column (Phenomenex®, Torrance, CA, USA, 2.1 × 100 mm, particle Ø = 2.6 µm, pore size 100 Å, surface area 200 m^2^/g), attached to an Ultivo® Triple Quadrupole LC/MS system controlled by MassHunter™ software (Agilent Technologies®, Inc., Santa Clara, USA), and consisting of a quaternary pump, column oven, autosampler, and a triple quadrupole mass detector, was used to quantify metabolites. 1 µL of the supernatant was injected onto the LC/MS system. The results were converted from ng/mL to ng/g of the wet weight of brain tissue.

### LPS and LBP analysis

Immediately prior to the analysis, plasma samples were allowed to thaw on ice. Both markers were analysed by means of enzyme-linked immunoassay, using commercially available kits according to the manufacturer’s instructions (LPS: SEB526Ge, Cloud-Clone® Corporation, USA; LBP: E-EL-M2686, Elabscience® Biotechnology, Inc., USA). The respective absorbances were read at a wavelength of 450 nm±10 nm for LPS and 450 nm±2 nm for LBP. The results were converted to and reported as ng/mL.

### Microbiome analysis

Microbial deoxyribonucleic acid (DNA) was extracted from faecal samples (approximately 0.25 g per sample) using a QIAamp® PowerFecal® DNA kit (QIAGEN®, Valencia, CA, USA). Extraction was performed as per the manufacturer’s instructions. The quality and quantity of extracted DNA were assessed by NanoDrop (ThermoFisher®, Johannesburg, South Africa). As a positive control, genomic DNA was extracted from mock bacterial community cells with equal colony-forming units from each of the 22 known species (HM-280, Biodefense and Emerging Infections Research Resources Repository [BEI]). Extracted genomic DNA was amplified by PCR in triplicate using primers targeting the V3–V4 hypervariable region of the 16S rRNA gene using the 357F/806R primers, as described previously (Dabee *et al*., 2021). Negative controls during DNA extraction and primary and secondary PCRs were included. Amplified libraries were purified using AMPure XP beads (Beckman Coulter®), and quantified by using Quant-iT dsDNA High Sensitivity Assay Kits (ThermoFisher®), pooled in equal molar amounts, and paired-end sequenced using a MiSeq Reagent Kit V3 (600-cycle, Illumina®).

### Statistical analysis

To analyse changes in nesting expression over time, as well as differences in TRP, its metabolites, relevant ratios, and plasma LPS and LBP, two-way analysis of variance (2-way ANOVA) was applied. In each instance, statistical significance of interactions, main effects, and pairwise comparisons (Bonferroni *post hoc*) was set at *p* <0.05. All pairwise comparisons were informed by calculations of Cohen’s *d* (with confidence intervals) to establish the magnitude of effect sizes (Cohen, 1988).

For microbiome data analysis, a Divisive Amplicon Denoising Algorithm (DADA) 2 (version 1.22.0) (Callahan *et al*., 2016) in R (version 4.2.0) (Gandrud, 2016) was first used to create an amplicon sequence variant (ASV) table. Reads were pre-processed using DADA 2 (Quast *et al*., 2012), learning error rates, and dereplication (eliminating redundant comparisons). Taxonomy was assigned using the SILVA reference database (v 138) as a reference (Quast *et al*., 2012). Downstream statistical analysis was performed with R (version 4.2.0) and RStudio (version 2022.7.1.554). The ASV table was transformed using centered log ratios (CLR). The iNEXT library (version 3.0.0) was used to compute alpha diversity (Chao1, Shannon entropy, and Simpson’s index). PERMANOVAs were calculated using the adonis2 function from the *vegan* package (version 2.6.4) using Aitchison distance with the formula ‘distance ∼ group treatment * nest building behaviour’, using 10 000 permutations. Principal component analysis was conducted after CLR transformation using R.

PICRUSt2 (version 2.4.1) (Douglas *et al*., 2020) was used to infer the genomic content from 16S rRNA gene data using the KEGG database (Kanehisa *et al*., 2016) as a reference. Inferred annotated genomes were used to compute the abundance of gut–brain modules (GBMs) (Valles-Colomer *et al*., 2019) using the OmixerRpm package (version 0.3.3) (Darzi *et al*., 2016). Differential abundance analysis of GBMs between groups was performed using the Tjazi R package (version 0.1.0.0) with the formula: ‘GBM ∼ group treatment * nest building behaviour + sequencing batch’ per each GBM (Bastiaanssen *et al*., 2023b; Bastiaanssen *et al*., 2023c). The variable sequencing batch was included to account for batch effects in the sequencing runs. *p* values were adjusted using the Benjamini–Hochberg FDR procedure, and a *q* value below 0.2 was deemed as significant (Bastiaanssen *et al*., 2023b; Bastiaanssen *et al*., 2023c). This procedure is also suggested for exploratory studies that aim to report results for later replication, where the risk for reporting false positive results is low (McDonald, 2014). For visualisation purposes, the CLR-transformed abundance of each GBM was standardised using the *Z*-score procedure, using the values across all the combinations of drug exposure and nest building behaviour. Host metabolites measurements were correlated with the inferred GBMs in the gut microbiome using Pearson’s rho correlation method. The anansi R package (version 0.5.0; Bastiaanssen *et al*. (2023a)) was used to define the subset of host metabolite-GBMs correlations to be tested for statistical significance.

## Results

### Nest building expression

#### Selection of mice for inclusion in the NNB and LNB cohorts

Of the 80 mice selected for the NNB and LNB groups, one mouse had to be excluded due to death prior to completing the nesting assessment. A significant negative correlation between the total nesting scores and between-day nesting variance of the remaining 79 mice was shown [*r*_s_(77) = −0.54, 95 CI: −0.69 to −0.36, *p* <0.0001] (Supplementary Fig. S2A). From this data, 24 LNB and 24 NNB mice were identified as explained in before. Subsequently, a significant difference between the median total nesting scores of the 24 NNB and 24 LNB mice, respectively, was shown (*U* = 576, *z* = 5.94, *p* <0.0001, data not shown).

#### Nesting response to drug exposure

With respect to the percentage change in the total nesting scores observed in NNB and LNB mice at the end of 5 weeks of either control or escitalopram exposure (Supplementary Fig. S2B), no significant two-way interaction was shown between phenotype and exposure [*F*(1,44) = 0.50, *p* = 0.48]. However, a significant main effect of phenotype was shown [*F*(1,44) = 13.2, *p* = 0.01]. Specifically, escitalopram-exposed LNB mice showed decreased nesting scores compared to those of NNB mice (−11.07 ± 32.73% vs. 71.8 ± 95.56%, *p* = 0.01, *d* = 1.12, 95 CI [−2.0–−0.3]). The same trend was observed for control-exposed LNB, compared to NNB mice, although this difference did not reach statistical significance (*p* = 0.09, *d* = 0.92, 95 CI [−1.8–−0.1]).

### TRP, metabolites, and ratios

**Figure 1.**
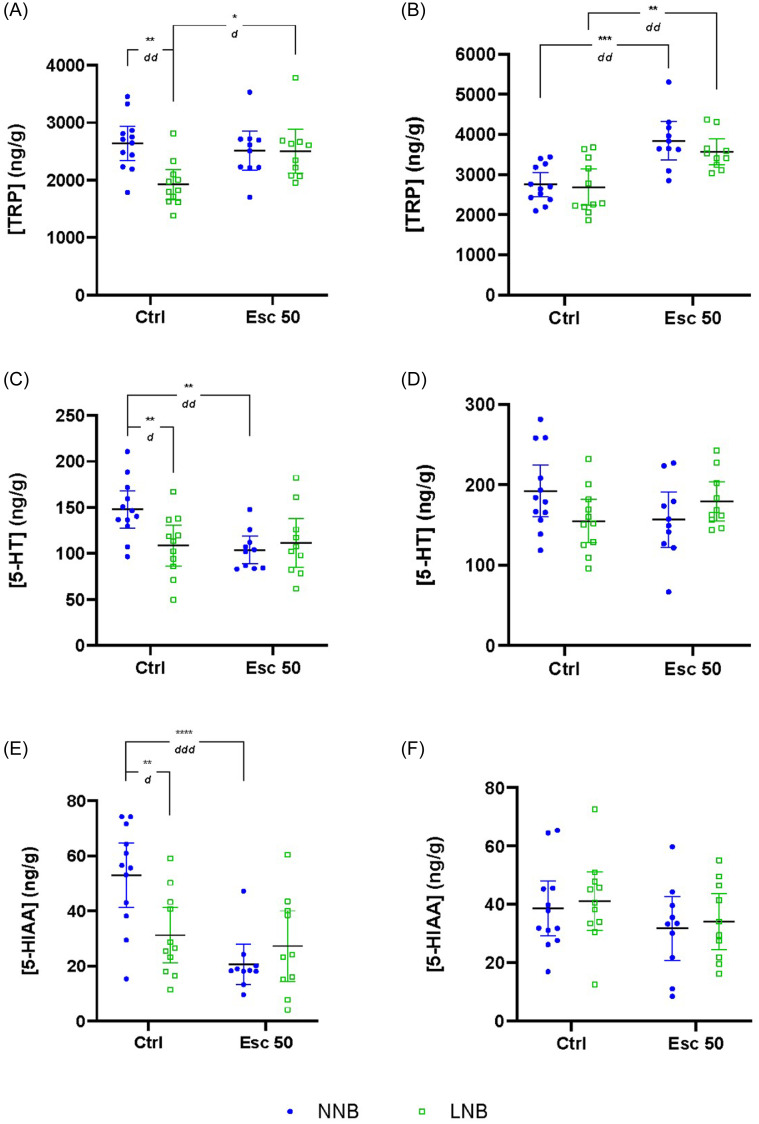
Differences in frontal-cortical and striatal tryptophan (TRP), serotonin (5-HT) and 5-hydroxyindoleacetic acid (5-HIAA) concentrations. Data analysed by means of 2-way ANOVA followed by Bonferroni’s multiple comparisons. Cohen’s *d* effect sizes as indicated: 0.8 < *d* < 1.3 < *dd* < 2 < *ddd*. ANOVA statistics represented in Table 1A–C. Data represented as mean ± 95% CI. **(A)** Frontal-cortical TRP, **p = 0.0015, *dd* = 1.59; *p = 0.0146, *d* = 1.2; **(B)** striatal TRP, ***p = 0.0001, *dd* = 1.46, **p = 0.0024, *dd* = 1.84; **(C)** frontal-cortical 5-HT, **p = 0.0099, *d* = 1.16; **p = 0.0044, *dd* = 1.54; **(D)** striatal 5-HT; **(E)** frontal-cortical 5-HIAA, **p = 0.0042, *d* = 1.24; ***p<0.0001, *d* = 2.04; **(F)** striatal 5-HIAA. NNB, normal nest building; LNB, large nest building.

**Figure 2.**
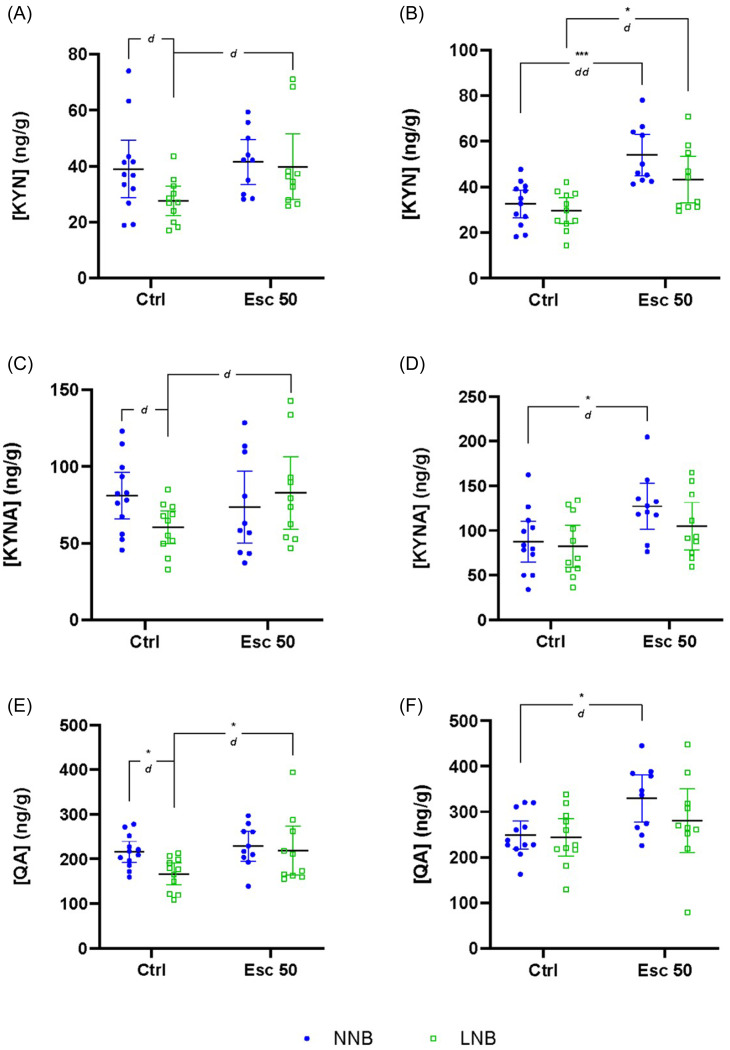
Differences in kynurenine (KYN), kynurenic acid (KYNA), and quinolinic (QA) concentrations. Data analysed by means of 2-way ANOVA followed by Bonferroni’s multiple comparisons. Cohen’s *d* effect sizes as indicated: 0.8 < *d* < 1.3 < *dd* < 2 < *ddd*. ANOVA statistics represented in Table 1D–F. Data represented as mean ± 95% CI. **(A)** Frontal-cortical KYN, *d* = 0.85; *d* = 0.93; **(B)** striatal KYN, ***p = 0.0002, *dd* = 1.85, *p = 0.0177, *d* = 1.13; **(C)** frontal-cortical KYNA, *d* = 0.97; *d* = 0.84; **(D)** striatal KYNA, *p = 0.0275, *d* = 1.06; **(E)** frontal-cortical QA, *p = 0.0493, *d* = 1.3; *p = 0.0463, *d* = 0.85; **(F)** striatal QA, *p = 0.0275, *d* = 1.28. NNB, normal nest building; LNB, large nest building.

**Figure 3.**
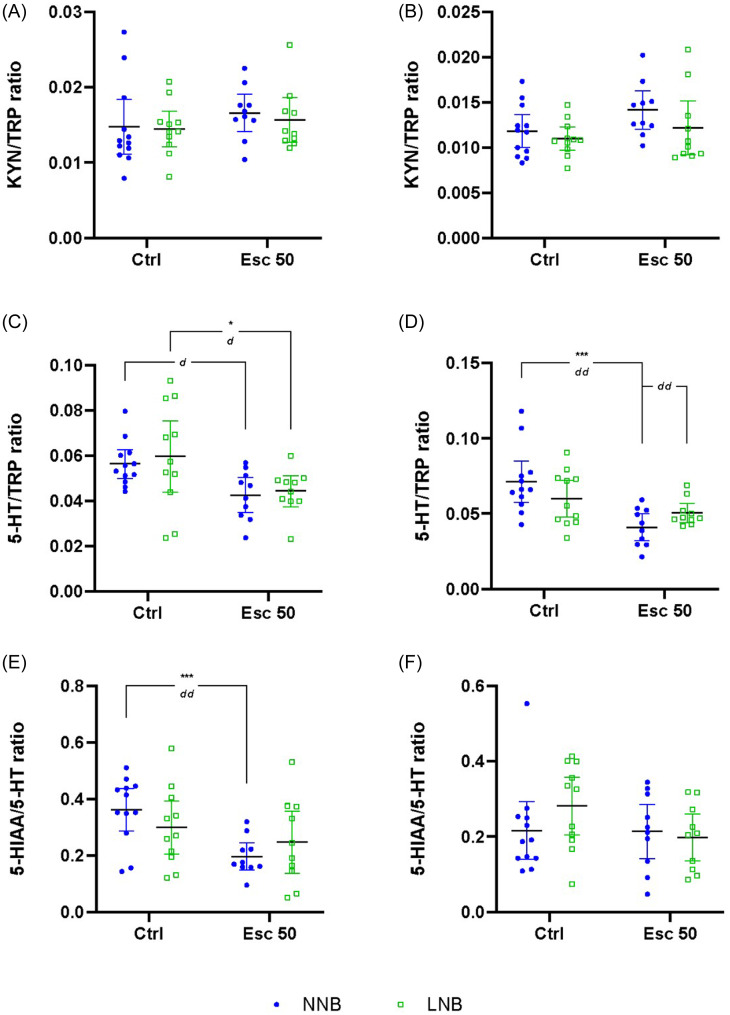
Differences in kynurenine/tryptophan (KYN/TRP), serotonin/tryptophan (5-HT/TRP) and 5-hydroxyindoleacetic acid/serotonin (5-HIAA/5-HT) ratios. Data analysed by means of 2-way ANOVA followed by Bonferroni’s multiple comparisons. Cohen’s *d* effect sizes as indicated: 0.8 < *d* < 1.3 < *dd* < 2 < *ddd*. ANOVA statistics represented in Table 2A–C. Data represented as mean ± 95% CI**. (A)** Frontal-cortical KYN/TRP; **(B)** striatal KYN/TRP; **(C)** frontal-cortical 5-HT/TRP, *d* = 1.28; *p = 0.0438, *d* = 0.81; **(D)** striatal 5-HT/TRP, ***p = 0.0002, *dd* = 1.61, *d* = 0.83; **(E)** frontal-cortical 5-HIAA/5-HT, **p = 0.0067, *dd* = 1.62; **(F)** striatal 5-HIAA/5-HT. NNB, normal nest building; LNB, large nest building.

**Figure 4.**
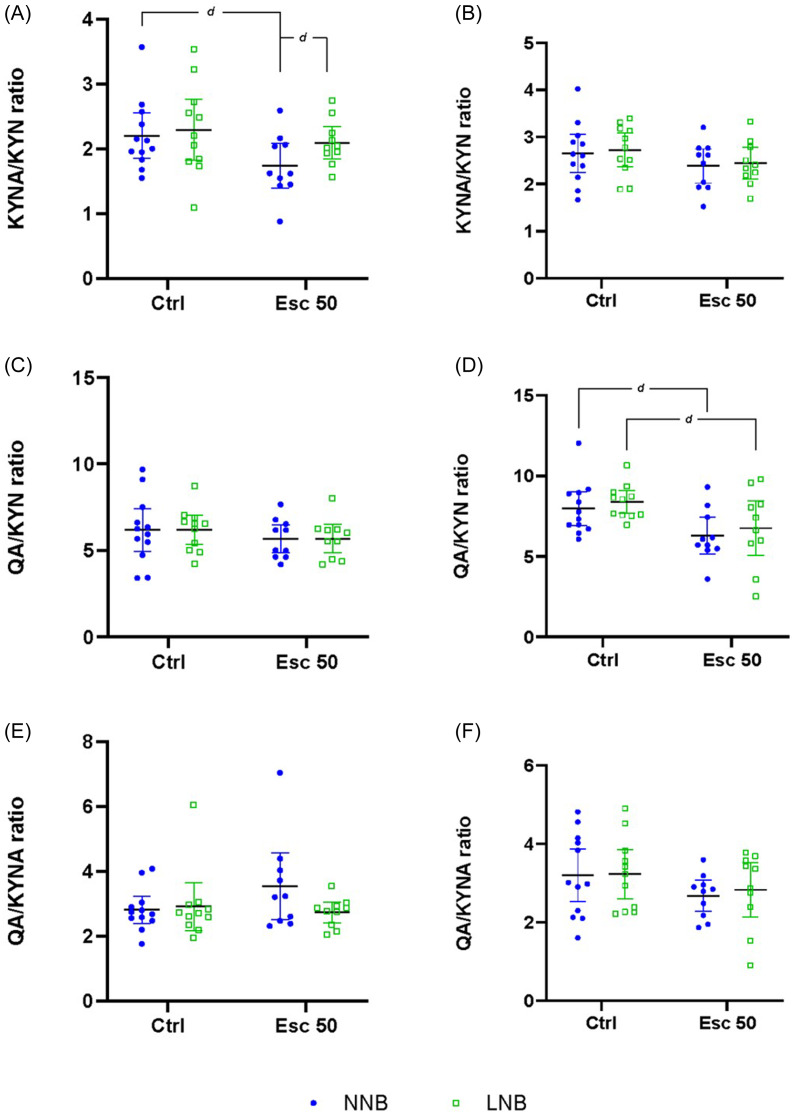
Differences in kynurenic acid/kynurenine (KYNA/KYN), quinolinic acid/kynurenine (QA/KYN) and quinolinic acid/kynurenic acid (QA/KYNA) ratios. Data analysed by means of 2-way ANOVA followed up with Bonferroni’s multiple comparisons. Cohen’s *d* effect sizes as indicated: 0.8 < *d* <1.3 < *d d* <2 < *ddd*. ANOVA statistics represented in Table 2D–F. Data represented as mean ± 95% CI. **(A)** Frontal-cortical KYNA/KYN, *d* = 0.86, *d* = 0.81; **(B)** striatal KYNA/KYN; **(C)** frontal-cortical QA/KYN; **(D)** striatal QA/KYN, *d* = 98, *d* = 0.86; **(E)** frontal-cortical QA/KYNA; **(F)** striatal QA/KYNA. NNB, normal nest building; LNB, large nest building.

**Figure 5.**
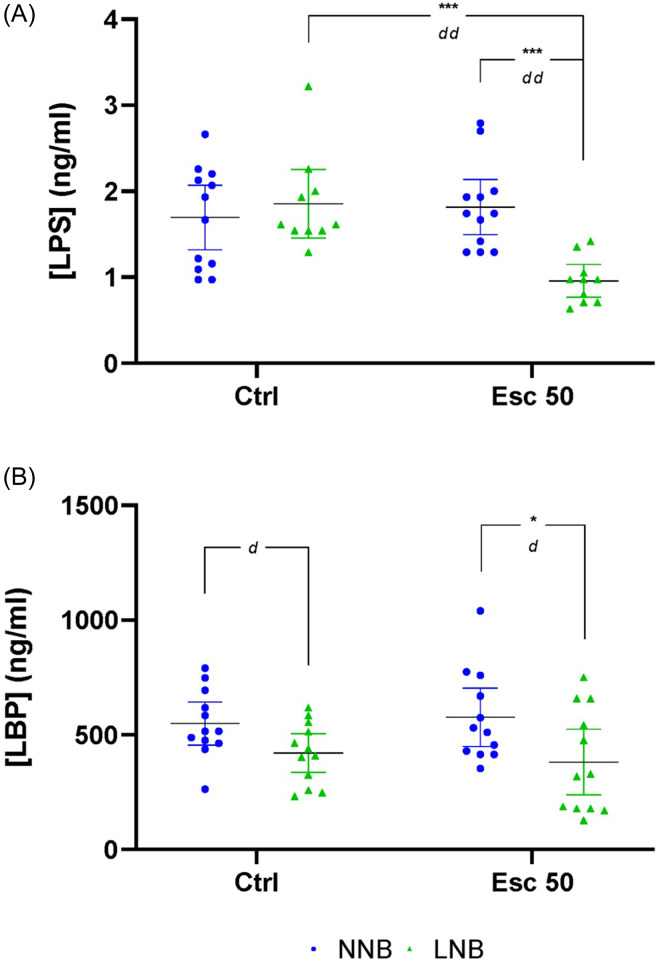
Differences in plasma lipopolysaccharide (LPS) and lipopolysaccharide binding protein (LBP) concentrations. Data analysed by means of 2-way ANOVA followed up with Bonferroni’s multiple comparisons. Cohen’s *d* effect sizes as indicated: 0.8 < *d* <1.3 < *d d* <2 < *ddd*. ANOVA statistics represented in Table 3A. Data represented as mean ± 95% CI. **(A)** LPS concentrations, ***p = 0.0005, *dd* = 1.97, ***p = 0.0005, *dd* = 1.99; **(B**) LBP concentrations, *d* = 0.88, *p = 0.022, *d* = 0.89. NNB, normal nest building; LNB, large nest building.

All descriptive data (mean ± SD) and statistical descriptors of the results are indicated in Tables 1–3 and Figs. 1–5, unless stated otherwise. Where attention to other comparisons is drawn, the statistical descriptors are provided in the text.

#### Concentrations of TRP and its metabolites

##### Frontal cortex

Significant two-way interactions between nesting phenotype and drug intervention were shown for TRP (**row A**), serotonin (**row B**), and 5-HIAA (**row C**), but not for KYN (**row D**), KYNA (**row E**), or QA (**row F**) (Figs. 1 and 2, Table 1). Further, whereas phenotype significantly influenced frontal-cortical TRP concentrations, drug intervention had a significant effect on serotonin, 5-HIAA, and QA concentrations.

Control-exposed LNB mice presented with significantly lower TRP concentrations compared to control-exposed NNB mice (Fig. 1A). This was reversed by escitalopram exposure, so that LNB mice presented with TRP concentrations akin to that of both control- (**NNB:** 2641.41 ± 467.27 vs. **LNB:** 2503.57 ± 527.19 ng/g, *p* >0.99) and escitalopram-exposed NNB mice.

Control-exposed LNB mice also presented with lower serotonin (Fig. 1C), 5-HIAA (Fig. 1E), and QA (Fig. 2E) concentrations compared to control-exposed NNB mice, while the same trend was observed for KYN and KYNA in both groups. With respect to the effect of drug exposure on frontal-cortical metabolite concentration, serotonin and 5-HIAA were reduced in NNB but remained unaltered in LNB mice after escitalopram exposure, whereas QA was increased in LNB, but not NNB mice.

##### Striatum

In the striata (Figs 1 and 2, Table 1), a significant two-way phenotype-exposure interaction was only shown for serotonin (**row B**). However, a significant main effect of drug exposure was demonstrated for TRP (**row A**), KYN (**row D**), KYNA (**row E**), and QA (**row F**).

Escitalopram exposure was associated with significantly increased total TRP (Fig. 1B) and KYN (Fig. 2B) concentrations of both NNB and LNB mice compared to their water-exposed counterparts, while KYNA (Fig. 2D) and QA (Fig. 2F) were significantly increased in NNB mice only.

#### Turnover ratios of TRP to its metabolites

##### Frontal cortex

To explore the directionality of TRP breakdown, turnover ratios of TRP and its downstream metabolites were analysed (Figs. 3 and 4, Table 2). No significant phenotype-exposure interactions were shown for any of the frontal-cortical turnover ratios calculated, that is, KYN/TRP (**row A**), serotonin/TRP (**row B**), 5-HIAA/serotonin (**row C**), KYNA/KYN (**row D**), QA/KYN (**row E**), and QA/KYNA (**row F**). However, a main effect of drug exposure was shown for serotonin/TRP and 5-HIAA/serotonin.

Specifically, escitalopram exposure was associated with decreased turnover of TRP to serotonin in LNB mice (Fig. 3C), with a similar trend also observed in NNB mice. Also, the conversion of serotonin to 5-HIAA was decreased in NNB, but not LNB mice (Fig. 3E).

##### Striatum

A significant two-way interaction between phenotype and exposure was shown with respect to the striatal serotonin/TRP (**row B**), but not for any of the other turnover ratios calculated. A significant main effect of drug exposure was shown for serotonin/TRP (**row B**) and QA/KYN (**row E**) (Figs. 3 and 4, Table 2).

Escitalopram significantly reduced the turnover of striatal TRP to serotonin in NNB mice (Fig. 3D), while there was a strong trend of a decreased KYN-to-QA turnover in both NNB and LNB mice.

### Plasma LPS and LBP

A significant phenotype-exposure interaction was shown for plasma LPS, but not LBP concentration (Fig. 5, Table 3, row A). Further both phenotype and exposure had a significant main effect on LPS concentration, while phenotype significantly influenced LBP concentrations.

Pairwise comparisons revealed a significant reduction in the LPS concentrations of escitalopram-exposed LNB compared to control-exposed LNB mice, while LBP concentrations were significantly lower in escitalopram-exposed LNB compared to escitalopram-exposed NNB mice.

### Gut microbiome analysis

#### Effects of nesting expression and escitalopram exposure on the taxonomic composition of the gut microbiome

**Figure 6.**
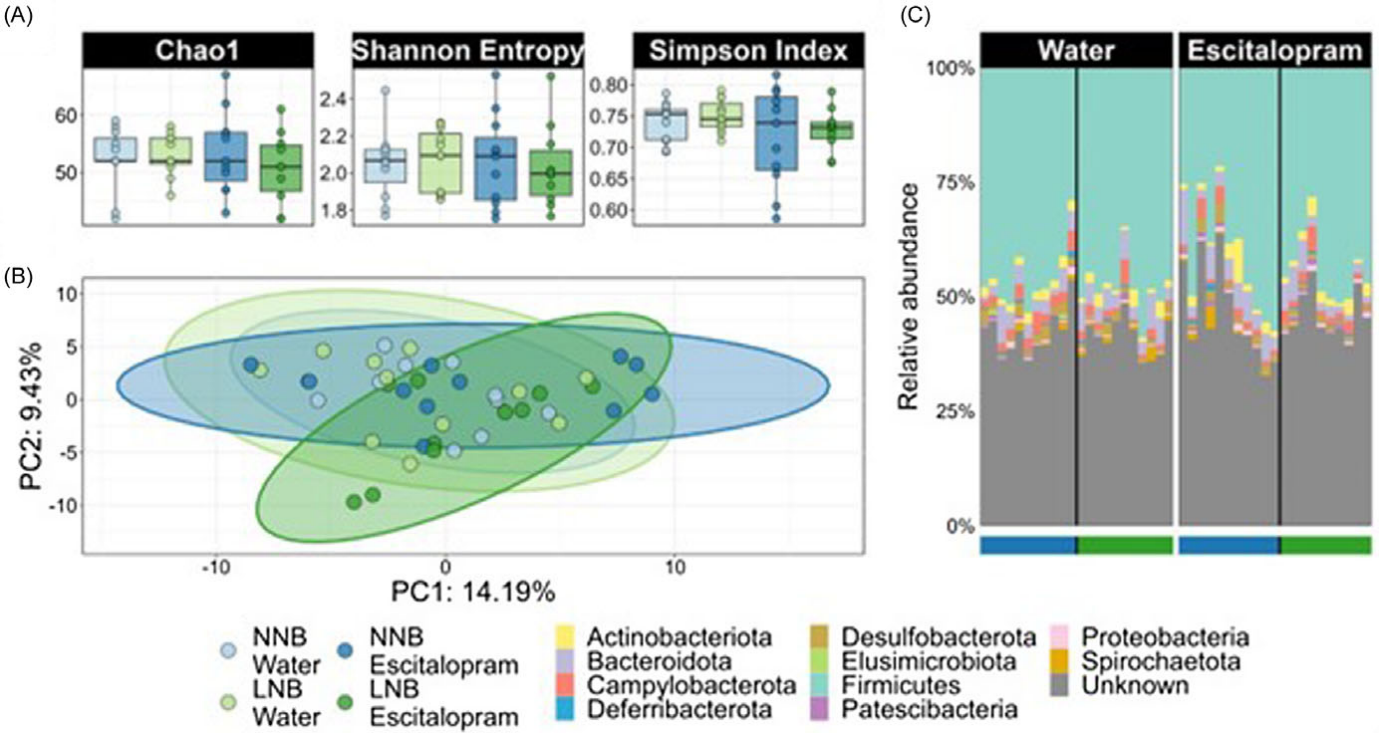
The taxonomic composition of the deer mice gut microbiome is stable in response to escitalopram exposure regardless of host basal nest building behaviour. (A) Alpha diversity of the gut microbial communities as reflected by Chao1, Shannon entropy, and Simpson’s index. (B) PCA of 16S data computed as Aitchison distance (Euclidean distances between samples with CLR-transformed abundances). (C) Stacked bar plot showing the taxonomic composition of the bacterial communities at the level of genus. NNB mice exposed to water are depicted in light blue, NNB mice exposed to escitalopram are depicted in dark blue, LNB mice exposed to water are depicted in light green, and LNB mice exposed to escitalopram are depicted in dark green. NNB: normal nest building; LNB: large nest building.

No statistical differences were detected between groups at the level of escitalopram exposure, nesting cohort, or their interaction for any of the three indices for intra-sample (alpha) diversity (Fig. 6A), that is, Chao1 [*F* (4, 38) = 0.51, *p* = 0.727], Shannon Entropy [*F* (4, 38) = 0.05, *p* = 0.994], and Simpson Index [*F* (4, 38) = 2.07, *p* = 0.104]. Significant effects on the between sample (beta) diversity were detected due to escitalopram exposure [pseudo-*F* = 1.58; *p* = 0.035], but not due to nesting cohort [pseudo-*F* = 1.11, *p* = 0.30] (Fig. 6B). However, the interaction between the two factors was also significant [pseudo-*F* = 1.54, *p* = 0.04]. Analysis of the relative gut bacterial abundances (Fig. 6C) revealed *Firmicutes* to be the most abundant phylum across all groups regardless of condition, with a mean relative abundance of 44.6% (SD = 8.8%). Unknown phyla across all groups accounted for a mean relative abundance of 43.6% (SD = 7.0%). The rest of the bacterial community was composed of nine other main bacterial phyla, indicating that neither escitalopram, nesting cohort, nor the interaction between these factors induced significant changes in the taxonomic composition at the phylum level.

#### Effects of escitalopram on the brain-modulatory potential of the gut microbiome depend on the baseline nest building behaviour of the host

**Figure 7.**
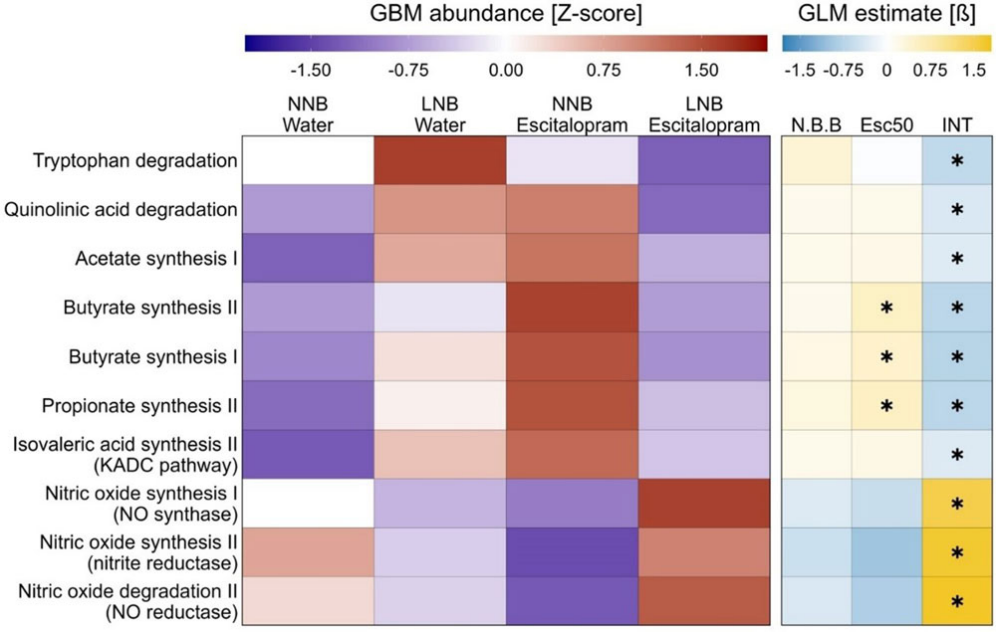
Effect of escitalopram on the inferred potential of the gut microbiome to metabolise neuroactive compounds depends on the basal nest building behaviour of the host. Left panel: standardised (Z-score) abundance of the inferred gut–brain modules (GBMs) for each condition. A higher intensity of purple depicts a lower inferred abundance of the relevant modules, whereas a higher intensity of red depicts a higher inferred abundance. Right panel: effect size (beta) estimate of the GLM for each factor applied in the formula. Positive values (yellow) indicate higher predicted genetic potential and negative values (blue) indicate the opposite for gut-brain communication per factor of the GLM. Data based on nest building behaviour (‘N.B.B’ column), escitalopram treatment (‘Esc50’ column), and their interaction (’INT’ column). Stars represent statistical significance after Benjamini–Hochberg correction (FDR; *p* < 0.05 and *q* < 0.2).

Additionally, the predicted neuroactive potential of the gut microbiome in deer mice was assessed in NNB and LNB deer mice exposed to either normal water or escitalopram using the GBM framework (Valles-Colomer *et al*., 2019). Changes in the inferred abundance of each GBM in the deer mouse gut microbiome were determined using GLMs (Fig. 7). Escitalopram exposure caused an increase in the inferred potential of the deer mouse gut microbiome to synthesise butyrate and propionate (Benjamini–Hochberg corrected *q* values <0.2). The same models, however, showed that the abundance of every inferred GBM of escitalopram-exposed mice responds differently depending on the baseline nesting expression of the host. Specifically, LNB mice exposed to escitalopram showed a significant decrease in the inferred potential to synthesise four different short-chain fatty acids (SCFAs) as well as a lower inferred potential to degrade TRP and its metabolite QA. Additionally, the same group showed a significant increase in its inferred potential to metabolise nitric oxide (NO) (Benjamini–Hochberg corrected *q* values <0.2). Notably, there was no significant relationship between the baseline nesting behaviour of mice and the abundance of any predicted GBM. Even so, our data suggest that the impact of escitalopram on the neuroactive potential of the gut microbiome is dependent on the baseline nesting expression of the host (Fig. 5).

#### Tissue-specific changes in host TRP metabolites correlate with the brain-modulatory potential of the gut microbiome

Finally, correlations between the inferred neuromodulatory potential of the gut microbiome and the relevant neuroactive compounds measured in different host tissues also suggest that these associations are dependent on the baseline nest building behaviour of mice, with escitalopram inducing changes on both their strength and magnitude. Indeed, statistically significant correlations driven by baseline nest building behaviour across different tissues are shown in Supplementary Fig. S3A, while correlations that showed a non-linear interaction between baseline nesting expression and drug exposure are shown in Supplementary Fig. S3B.

### Discussion

The main findings of this work are that (1) LNB, but not NNB, decreased after escitalopram exposure; (2) LNB is associated with reduced frontal-cortical TRP concentrations and hyposerotonergia; (3) the latter is unrelated to altered KYN pathway activity or inflammatory involvement, as reflected by plasma LPS concentration; and (4) although escitalopram exposure associated with changes in the microbiota, the effects of escitalopram on the inferred neuromodulatory potential of the gut microbiota depend on the baseline nesting expression of the host.

Our data pertaining to the response of LNB over time, irrespective of drug intervention, highlight unique psychobiological underpinnings in the naturalistic expression of NNB and LNB. Given that the nesting expression of NNB, but not LNB mice, inflated over time in both control- and escitalopram-exposed mice, the present findings are in line with previous results from our lab (Saaiman *et al*., 2023). Previous results also showed that chronic escitalopram administered at the same dose and for a similar duration prevented nesting inflation in LNB mice (Wolmarans *et al*., 2016; de Brouwer *et al*., 2020a). Neurobiological separation between NNB and LNB is also evidenced by frontal-cortical hyposerotonergia in LNB, compared to NNB mice (Fig. 1C), a finding that is also consistent with previous work indicating altered serotonergic processes in compulsive-like behavioural rigidity (Greene-Schloesser *et al*., 2011; Wolmarans *et al*., 2022). This finding was echoed by lower TRP (Fig. 1A) and 5-HIAA (Fig. 1E) concentrations in parallel with unaltered TRP-to-serotonin (Fig. 3C) and TRP-to-KYN (Fig. 3A) turnover or a change in the rate of KYN breakdown to either KYNA or QA (Fig. 4A and C). Thus, the lower LNB-associated frontal-cortical serotonin concentrations are likely related to an overall lower systemic TRP availability. Nevertheless, and importantly, escitalopram exposure restored frontal-cortical TRP in LNB mice without affecting serotonin and 5-HIAA concentrations. Thus, we highlight a potential neurobiological mechanism of action of escitalopram on deer mouse behaviour that is unrelated to direct serotonergic modulation. This finding is informative, especially considering that escitalopram also blunted the inferred TRP degradation potential in the gut microbiome of escitalopram-exposed, compared to water exposed LNB, but not NNB mice (Fig. 7). It is thus plausible that while this effect contributed to the increased frontal-cortical and striatal TRP availability in LNB mice, the impact of escitalopram on the gut microbiota (see below) and the associated behavioural outcomes in LNB mice, could be founded on another, yet unresolved gut-brain mechanism that does not only depend on changes in central serotonin concentrations. The increase in central TRP availability shown here may be a proxy for such a mechanism. This notion is especially intriguing, given the overall impact of escitalopram on the inferred functional potential of the gut microbiome in LNB versus NNB deer mice (see later). While frontal-cortical TRP concentrations in NNB mice were unaffected by escitalopram, a significant increase in striatal concentrations was observed. However, in NNB mice, these observations paralleled a decreased turnover to serotonin and 5-HIAA (Fig. 1C and E), without impacting the behavioural output in this cohort (Supplementary Fig. 2B). Further, the sampling technique used here, that is, tissue homogenisation, mostly allows for interpreting total serotonin concentrations in terms of central storage capacity only (Hale and Lowry, 2011). Taking these lines of thought together, we conclude that while LNB, but not NNB, is linked to an overall hyposerotonergic profile at baseline, the LNB phenotype and its response to SSRI exposure may be founded upon more complex interactions between central and peripheral mechanisms than merely on serotonergic modulation. This conclusion is consistent with clinical literature indicating OCD to be associated with long-term ‘atypical’ profiles of serotonergic activity, as opposed to hyposerotonergia *per se* (Goddard *et al*., 2008).

Expanding on the above, a common mechanism proposed to underlie reduced brain serotonin availability relates to an increase in TRP-to-KYN turnover (Marx *et al*., 2020). However, despite the purported presence of immunological correlates in OCD (see Szechtman *et al*. (2020) for review) and ASD (Meltzer and Van de Water, 2017), altered TRP-to-KYN turnover has, according to our knowledge, not been shown before, whereas the present data also do not reveal such a mechanism underlying the expression of LNB. Our data also do not show a role for glutamatergic dysregulation as reflected by QA and KYNA modulation, even though glutamatergic perturbations may be an underlying mechanism in some patients (Marazziti *et al*., 2018a). Interestingly, although not significant, we show an increase in the inferred potential of the LNB microbiome to synthesise and break down NO. NO synthesis is directly linked to glutamatergic signalling via its activation of the NMDA receptor (Brown and Bal-Price, 2003). However, the potential impact of dysregulated NO metabolism in the deer mouse remains to be explored, although a possible role for NO has been implied (Krass *et al*., 2010) using marble burying as a behavioural outcome akin to OCD-like symptoms (de Brouwer *et al*., 2018). Considering that LNB is entirely naturalistic and that early- and later-life perturbations in KYN pathway activity can have long-lasting effects on neuropsychiatric outcomes (Pocivavsek *et al*., 2012; Alexander *et al*., 2013; Pocivavsek *et al*., 2014), notably so in the type of disorders referred to here (Rickards *et al*., 1996; Hoekstra *et al*., 2007), the same was not shown with respect to the expression of LNB. This is important because elevations in QA are also associated with heightened NMDA receptor activation (and increased NO release), a potential mechanism that may underlie striatal activation (Schwarcz and Köhler, 1983; Nakanishi, 1992). Rather, we propose that since escitalopram increased the striatal concentrations of KYN, KYNA, and QA in NNB mice, an optimal homeostatic state likely already prevailed in terms of serotonin synthesis. Therefore, TRP was merely shunted along the KYN pathway without affecting the QA/KYNA ratio (Fig. 4E and F). Last, that most of the reported differences between NNB and LNB mice were demonstrated in frontal-cortical as opposed to striatal tissue provides putative proof-of-concept that LNB is founded upon dysfunctional psychobiological processes related to higher order goal-directed action-outcome planning, rather than striatal processes that govern and facilitate the motor execution of such planned behaviours (Bourne *et al*., 2012; Yager *et al*., 2015).

We have previously shown that LNB mice present with a distinct, potentially pro-inflammatory, gut microbiota profile compared to NNB mice (Scheepers *et al*., 2020). Since peripheral inflammatory states – notably also in the gut – are known to bolster TRP turnover along the KYN pathway, we hypothesised that LNB mice might show a distinct microbiota-related pro-inflammatory profile as reflected by increased plasma LPS and LBP concentrations (Rosadini and Kagan, 2017; Just *et al*., 2021). It was noteworthy that while NNB and LNB mice presented with similar plasma LPS concentrations at baseline (Fig. 5), escitalopram exposure resulted in a significant and meaningful reduction in the plasma LPS concentrations of LNB, but not NNB mice. This observation further supports the view that escitalopram uniquely impacted the gut microbiota of LNB expressing deer mice and confirms LNB as a neurobiologically distinct phenotype, adequately identified by means of bidirectional separation. Apart from escitalopram blunting the inferred TRP degradation potential of the microbiota colonising the LNB gut, predicted functional analysis of the gut microbiota also sought to explore changes in inflammation-related GBMs in LNB versus NNB mice. Contrary to the working hypothesis, escitalopram elicited a higher inferred potential to synthesise the anti-inflammatory SCFAs, acetate, butyrate, isovaleric acid, and propionate in NNB mice, compared to the group exposed to water only. Interestingly, this trend was reversed in LNB mice, where escitalopram reduced the inferred potential of the microbiome to synthesise SCFAs (Fig. 7). Attention should be drawn to the fact that escitalopram trended towards modulating the relevant GBMs in LNB mice to be mostly akin to that of water exposed NNB, that is, normal mice (Fig. 7), potentially highlighting a ‘restorative’ effect on the gut microbiota of LNB mice, which again could be proxy for another biological anti-compulsive-like process triggered in LNB mice only. Interestingly, the inferred potential of the gut microbiome to synthesise propionate and butyrate were the only pathways known to affect the gut-brain axis that showed statistically significant differences between water and escitalopram-exposed mice, irrespective of the baseline nest building behaviour of the host (Fig. 7). This may further emphasise other potentially brain-modulatory effects of the drug. It can be concluded that the parallel reduction in plasma LPS concentrations and the blunted inferred potential of the gut microbiota to metabolise TRP in escitalopram-exposed LNB mice is not related to changes in the production of anti-inflammatory SCFAs and is thus founded upon a mechanism that is yet unknown.

In addition to the functional analysis, we also performed a taxonomic description of the gut microbiome of these mice. As opposed to our earlier work (Scheepers *et al*., 2020), no significant effect of baseline nesting behaviour on the beta-diversity was shown in the present study. While this is surprising, multigenerational shifts in the gut microbiota composition of the same-species mice are a common occurrence. Indeed, such multifactorial influences include host genotype (Benga *et al*., 2024) and rearing condition (Nguyen *et al*., 2015). Since the deer mice used in our studies are bred wild type, shifts in the gut microbiota composition of mice used in the present investigation, compared to mice studied previously, can be expected. However, the application of different databases for the taxonomic annotation of the 16S data (SILVA, version 138 vs. Ribosomal Database Project used earlier) may also be a cause. Since SILVA covers more taxa and was updated more recently, it is currently the accepted standard in the field for the analysis of 16S rRNA gene sequencing data. Nevertheless, the unique response of the LNB gut microbiome to escitalopram reiterates temporal uniqueness among the NNB and LNB gut microbiome that is useful for studies of gut-brain relationships.

We also explored the effects of escitalopram on the taxonomic composition of the gut bacterial communities of NNB and LNB mice. In this respect, the effect of escitalopram on the bacterial community composition was dependent on the baseline nesting behaviour of the host (Fig. 6B). This change highlights a minor effect of escitalopram on the overall composition of gut bacterial microbiota. However, even if the effects of escitalopram on the gut bacteria are small in terms of phylum composition (Fig. 6C), escitalopram induced important changes in the inferred functions of the microbiome community, which were phenotype dependent. This finding is supported by a previous clinical study on the broad effects of psychotropic drugs (e.g. antidepressants) on the microbiome, which similarly showed affected microbial TRP metabolism post-treatment (Tomizawa *et al*., 2020). Moreover, changes in the gut microbiome could mediate other differences detected in the NNB and LNB gut-brain axis. For instance, escitalopram restored correlations between plasma serotonin and the potential of the microbiome to synthesise molecules known to modulate the gut barrier integrity, that is, acetate (Schälter *et al*., 2022), isovaleric acid (Ghosh *et al*., 2021), and the neurotransmitter glutamate, the latter evinced by the predicted synthesis and breakdown of NO (Supplementary Fig. 3B). Still, other associations between the abundance of TRP catabolites in different host body sites and the predicted neuroactive potential of the gut microbiome remained unaltered after escitalopram administration (Supplementary Fig. 3A). Collectively, these findings highlight the relevance of functional analyses of the gut microbiome when assessing the effects of external interventions on the biobehavioural outputs of animals used for translational research.

Some study limitations deserve emphasis. First, in terms of behavioural separation, the 24 mice included in the LNB and NNB cohorts were randomised in terms of nesting score between the two exposure groups. In other words, mice with varying nesting scores, albeit selected for inclusion in the same nesting phenotype, were grouped together in the escitalopram- and control-exposure groups, respectively. Future studies that employ larger sample sizes, also allowing for the study of sex-dependent effects, may benefit from a correlational analysis of nesting behaviour and its association with different biological parameters. To this end, clustering mice of the same sex showing similar baseline nesting scores and neurobiological profiles in the same experimental groups will be informative. Second, a post-drug-exposure LPS challenge in escitalopram-exposed NNB and LNB mice would have been valuable to trace a direct relationship between LPS and nesting expression. For the same reason, a focused investigation of inflammatory processes, that is, IDO and TDO activity, cytokine expression, and cortisol release, would have been beneficial. Lastly, the microbiome of *P. maniculatus bairdii* is not a well characterised ecosystem yet. It is therefore likely underrepresented in the databases used for taxonomic identification and inferred functional potential. Consequently, metabarcoding approaches for the taxonomic and inferred functional description of this community might be limited and partial.

## Conclusion

The current investigation of LNB in deer mice as a model of naturalistic behavioural rigidity aimed to determine whether LNB is characterised by alterations in inflammatory, TRP, and gut microbiota profiles. We found that LNB mice have lower baseline TRP and serotonin concentrations, without showing inflammation-related changes in the turnover of neuroactive metabolites. Escitalopram exposure further led to increased systemically available TRP, which was associated with a blunted inferred TRP degradation potential of the gut microbiota. Taken together, our data are in line with evidence of altered serotonergic mechanisms in OCD and highlight the complexity of escitalopram’s actions in the model system. The data here support the role of alterations in serotonergic mechanisms, inflammatory processes, and gut microbiome content in compulsive-like behavioural rigidity. There was, however, no evidence for alterations in KYN pathways. Our results also highlight the importance of gut-brain crosstalk mechanisms at the level of TRP metabolism in the spontaneous development of such behaviour. Collectively, this body of research may contribute to our understanding of the naturalistic mechanisms that may underlie and perpetuate psychiatric disorders characterised by rigidity (e.g. OCD).

**Table 1.** Descriptive statistics of frontal-cortical and striatal TRP, serotonin (5-HT), 5-HIAA, KYN, KYNA, and QA concentrations

		*PFC*					*STR*				
		Mean ± SD (ng/g)	*F*	*p*	*d*	CI*d*	Mean ± SD (ng/g)	*F*	*p*	*d*	CI*d*
**(A) TRP**		***Interaction***					***Interaction***				
	Drug exposure × Phenotype		(1,39) = 6.06	0.0183*				(1, 39) = 0.37	0.5487		
		***Main Effects***					***Main Effects***				
	Drug exposure		(1, 39) = 2.50	0.1220				(1, 39) = 3.13	<0.0001****		
	Phenotype		(1, 39) = 6.47	0.0150*				(1, 39) = 0.94	0.3381		
		***Pairwise Comparisons***					***Pairwise Comparisons***				
	***NNB vs. LNB***										
	Ctrl	2641.41 ± 467.27 vs. 1925.18 ± 393.70		0.0015**	**1.59**	−2.59 — −0.698	2750.2 ± 467.27 vs. 2685.95 ± 672.08		>0.9999	0.12	−0.93 — −0.71
	Esc	2515.37 ± 480.53 vs. 2503.57 ± 527.19		>0.9999	0.02	−0.9 — −0.85	3841.83 ± 677.37 vs. 3564.59 ± 455.95		0.5761	0.46	−0.93 — −0.72
	***Ctrl vs. Esc***										
	NNB	2641.41 ± 467.27 vs. 2515.37 ± 480.53		>0.9999	0.26	−1.11 — −0.58	2750.2 ± 467.27 vs. 3841.83 ± 677.37		0.0001***	**1.84**	0.87 — 2.92
	LNB	1925.18 ± 393.70 vs. 2503.57 ± 527.19		0.0146*	**1.2**	0.296 — 2.182	2685.95 ± 672.08 vs. 3564.59 ± 455.95		0.0024**	**1.46**	0.52 — 2.48
**(B) 5-HT**		***Interaction***					***Interaction***				
	Drug exposure × Phenotype		(1, 39) = 5.98	0.0191*				(1, 39) = 5.00	0.0312*		
		***Main Effects***					***Main Effects***				
	Drug exposure		(1, 39) = 4.61	0.0381*				(1, 39) = 0.176	0.6771		
	Phenotype		(1, 39) = 2.63	0.1129				(1, 39) = 0.296	0.5892		
		***Pairwise Comparisons***					***Pairwise Comparisons***				
	***NNB vs. LNB***										
	Ctrl	147.88 ± 31.93 vs. 108.76 ± 33.03		0.0099**	**1.16**	−2.09 — −0.3	192.36 ± 50.71 vs. 154.92 ± 40.14		0.097	0.79	−1.66 — −0.05
	Esc	103.70 ± 21.13 vs. 111.64 ± 37.16		>0.999	0.25	−2.09 — −0.4	156.60 ± 48.04 vs. 179.38 ± 34.11		0.5091	0.52	−0.36 — 1.43
	***Ctrl vs. Esc***										
	NNB	147.88 ± 31.93 vs. 103.70 ± 21.13		0.0044**	**1.54**	−2.56 — −0.61	192.36 ± 50.71 vs. 156.60 ± 48.04		0.1306	0.7	−1.58 — 0.15
	LNB	108.76 ± 33.03 vs. 111.64 ± 37.16		>0.9999	0.08	−0.78 — 0.94	154.92 ± 40.14 vs. 179.38 ± 34.11		0.4225	0.63	−0.24 — 1.53

		*PFC*								*STR*							
		**Mean ± SD** **(ng/g)**	***F***		***p***		***d***		**CI*d***	**Mean ± SD** **(ng/g)**	***F***		***p***		***d***		**CI*d***
**(C) 5-HIAA**		***Interaction***								***Interaction***							
	Drug exposure × Phenotype		(1, 39) = 8.59		0.0056**						(1, 39) = 1.30^-5^		0.9971				
		***Main Effects***								***Main Effects***							
	Drug exposure		(1, 39) = 14.0		0.0006***						(1, 39) = 2.42		0.1281				
	Phenotype		(1, 39) = 2.44		0.1266						(1, 39) = 0.287		0.5950				
		***Pairwise Comparisons***								***Pairwise Comparisons***							
	***NNB vs. LNB***																
	Ctrl	53.05 ± 18.48 vs. 31.23 ± 15.12			0.0042**		**1.24**		−2.18 — −0.37	38.68 ± 14.68 vs. 41.09 ± 14.91			>0.9999		0.16		−0.66 — 0.98
	Esc	20.63 ± 10.11 vs. 27.29 ± 17.87			0.709		0.44		−0.44 — 1.34	31.76 ± 15.25 vs. 34.13 ± 13.46			>0.9999		0.16		−0.72 — 1.04
	***Ctrl vs. Esc***																
	NNB	53.05 ± 18.48 vs. 20.63 ± 10.11			<0.0001****		**2.04**		−3.17 — 1.04	38.68 ± 14.68 vs. 31.76 ± 15.25			0.5493		0.45		−1.31 — 0.39
	LNB	31.23 ± 15.12 vs. 27.29 ± 17.87			>0.9999		0.23		−1.1 — 0.62	41.09 ± 14.91 vs. 34.13 ± 13.46			0.5641		0.47		−1.35 — 0.39
**(D) KYN**		***Interaction***								***Interaction***							
	Drug exposure × Phenotype		(1, 39) = 1.39	0.2458							(1, 39) = 1.23	0.2750					
		***Main Effects***								***Main Effects***							
	Drug exposure		(1, 39) = 3.24	0.0794							(1, 39) = 25.4	<0.0001****					
	Phenotype		(1, 39) = 2.51	0.1213							(1, 39) = 3.92	0.0549					
		***Pairwise Comparisons***								***Pairwise Comparisons***							
	***NNB vs. LNB***																
	Ctrl	39.00 ± 16.19 vs. 27.62 ± 7.86		0.0997		**0.85**		−1.73 — −1.01		32.6 ± 9.54 vs. 29.57 ± 8.49		>0.9999		0.32		−1.16 — 0.49	
	Esc	41.52 ± 11.16 vs. 39.56 ± 16.34		>0.9999		0.11		−1.00 — 0.76		53.95 ± 12.8 vs. 43.23 ± 14.26		0.0825		0.76		−1.7 — 0.13	
	***Ctrl vs. Esc***																
	NNB	39.00 ± 16.19 vs. 41.52 ± 11.16		>0.9999		0.17		−0.66 — 1.02		32.6 ± 9.54 vs. 53.95 ± 12.8		0.0002***		**1.85**		0.88 — 2.93	
	LNB	27.62 ± 7.86 vs. 39.56 ± 16.34		0.0872		**0.93**		0.05 — 1.87		29.57 ± 8.49 vs. 43.23 ± 14.26		0.0177*		**1.13**		0.23 — 2.1	

		*PFC*					*STR*				
		**Mean ± SD** **(ng/g)**	***F***	***p***	***d***	**CI*d***	**Mean ± SD** **(ng/g)**	***F***	***p***	***d***	**CI*d***
**(E) KYNA**		***Interaction***					***Interaction***				
	Drug exposure × Phenotype		(1, 39) = 3.28	0.0780				(1, 39) = 0.626	0.4336		
		***Main Effects***					***Main Effects***				
	Drug exposure		(1, 39) = 0.814	0.3724				(1, 39) = 7.96	0.0075**		
	Phenotype		(1, 39) = 0.461	0.5011				(1, 39) = 1.58	0.2166		
		***Pairwise Comparisons***					***Pairwise Comparisons***				
	***NNB vs. LNB***										
	Ctrl	80.91 ± 23.95 vs. 60.37 ± 15.89		0.1517	**0.97**	−1.86 — −0.12	87.57 ± 35.9 vs. 82.46 ± 34.79		>0.9999	0.14	−0.96 — 0.68
	Esc	71.42 ± 32.79 vs. 82.76 ± 33.12		0.8877	0.27	−0.6 — 1.16	104.77 ± 37.03 vs. 127.26 ± 36.04		0.3387	0.59	−1.51 — 0.29
	***Ctrl vs. Esc***										
	NNB	80.91 ± 23.95 vs. 71.42 ± 32.79		>0.9999	0.26	−1.11 — 0.58	87.57 ± 35.9 vs. 127.26 ± 36.04		0.0275*	**1.06**	0.19 — 2.00
	LNB	60.37 ± 15.89 vs. 82.76 ± 33.12		0.13	**0.84**	−0.03 — −1.77	82.46 ± 34.79 vs. 104.77 ± 37.03		0.3264	0.6	−0.27 — 1.49
**(F) QA**		***Interaction***					***Interaction***				
	Drug exposure × Phenotype		(1, 39) = 1.63	0.2093				(1, 39) = 1.01	0.3222		
		***Main Effects***					***Main Effects***				
	Drug exposure		(1, 39) = 4.41	0.0422*				(1, 39) = 7.28	0.0103*		
	Phenotype		(1, 39) = 3.66	0.0631				(1, 39) = 1.54	0.2215		
		***Pairwise Comparisons***					***Pairwise Comparisons***				
	***NNB vs. LNB***										
	Ctrl	216.00 ± 36.93 vs. 166.26 ± 36.89		0.0493*	**1.300**	−2.25 — −0.42	248.79 ± 48.75 vs. 243.57 ± 61.33		>0.9999	0.09	−0.91 — 0.73
	Esc	228.85 ± 46.87 vs. 218.93 ± 76.73		>0.9999	0.15	−1.03 — 0.72	329.44 ± 72.38 vs. 280.51 ± 98.36		0.2655	0.54	−1.46 — 0.34
	***Ctrl vs. Esc***										
	NNB	216.00 ± 36.93 vs. 228.85 ± 46.87		>0.9999	0.30	−0.54 — 1.15	248.79 ± 48.75 vs. 329.44 ± 72.38		0.0275*	**1.28**	0.39 — 2.25
	LNB	166.26 ± 36.89 vs. 218.93 ± 76.73		0.0463*	**0.85**	−0.02 — 1.78	243.57 ± 61.33 vs. 280.51 ± 98.36		0.3264	0.44	−0.42 — 1.32

TRP, tryptophan; 5-HIAA, 5-hydroxyindoleacetic acid; KYN, kynurenine; KYNA, kynurenic acid; QA, quinolinic acid; NNB, normal nest building; LNB, large nest building; PFC, prefrontal cortex; STR, striatum.

**Table 2.** Descriptive statistics of frontal-cortical and striatal *KYN/TRP, 5-HT/TRP, 5-HIAA/5-HT, KYNA/KYN, QA/KYN,* and *QA/KYNA ratios*

		*PFC*					*STR*				
		Mean ± SD	*F*	*p*	*d*	CI*d*	Mean ± SD	*F*	*p*	*d*	CI*d*
**(A) KYN/TRP**		***Interaction***					***Interaction***				
	Drug exposure × Phenotype		(1, 39) = 0.0630	0.8032				(1, 39) = 0.382	0.5400		
		***Main Effects***					***Main Effects***				
	Drug exposure		(1, 39) = 1.28	0.2646				(1, 39) = 3.63	0.0640		
	Phenotype		(1, 39) = 0.206	0.6526				(1, 39) = 2.21	0.1450		
		***Pairwise Comparisons***					***Pairwise Comparisons***				
	***NNB vs. LNB***										
	Ctrl	0.02 ± 0.01 vs. 0.014 ± 0.003		>0.9999	0.06	−0.87 — −0.76	0.01 ± 0.003 vs. 0.01 ± 0.002		>0.9999	0.32	−1.14 — 0.5
	Esc	0.02 ± 0.003 vs. 0.02 ± 0.004		>0.9999	0.24	−1.12 — 0.64	0.01 ± 0.003 vs. 0.01 ± 0.004		0.3156	0.52	−1.43 — 0.36
	***Ctrl vs. Esc***										
	NNB	0.02 ± 0.01 vs. 0.017 ± 0.003		0.6586	0.37	−0.47 — 1.23	0.01 ± 0.003 vs. 0.014 ± 0.003		0.1581	0.08	−0.08 — 1.67
	LNB	0.01 ± 0.003 vs. 0.02 ± 0.004		>0.9999	0.3	−0.56 — 1.17	0.01 ± 0.002 vs. 0.01 ± 0.004		0.7454	0.36	−0.49 — 1.24
**(B) 5-HT/TRP**		***Interaction***					***Interaction***				
	Drug exposure × Exposure		(1, 39) = 0.03	0.8605				(1, 39) = 4.22	0.0468*		
		***Main Effects***					***Main Effects***				
	Drug exposure		(1, 39) = 10.5	0.0025**				(1, 39) = 15.5	0.0003***		
	Phenotype		(1, 39) = 0.33	0.5696				(1, 39) = 0.0345	0.8536		
		***Pairwise Comparisons***					***Pairwise Comparisons***				
	***NNB vs. LNB***										
	Ctrl	0.06 ± 0.01 vs. 0.06 ± 0.02		>0.999	0.18	−0.63 — 1.01	0.07 ± 0.02 vs. 0.06 ± 0.02		0.2178	0.54	−1.39 — 0.28
	Esc	0.04 ± 0.01 vs.0.04 ± 0.01		>0.999	0.17	−0.71 — 1.05	0.04 ± 0.01 vs. 0.05 ± 0.01		0.4182	**0.83**	−0.06 — 1.78
	***Ctrl vs. Esc***										
	NNB	0.06 ± 0.01 vs. 0.04 ± 0.01		0.07	**1.28**	−2.25 — −0.38	0.07 ± 0.02 vs. 0.041 ± 0.012		0.0002***	**1.61**	−2.64 — −0.67
	LNB	0.06 ± 0.03 vs. 0.04 ± 0.01		0.0438*	**0.81**	−1.73 — 0.06	0.06 ± 0.02 vs. 0.05 ± 0.01		0.3871	0.63	−1.53 — 0.24
	Drug exposure × Phenotype		(1, 39) = 2.26	0.1410				(1, 39) = 1.54	0.2218		
		***Main Effects***					***Main Effects***				
	Drug exposure		(1, 39) = 8.25	0.0066**				(1, 39) = 1.69	0.2011		
	Phenotype		(1, 39) = 0.0259	0.8729				(1, 39) = 0.563	0.4576		
		***Pairwise Comparisons***					***Pairwise Comparisons***				
	***NNB vs. LNB***										
	Ctrl	0.36 ± 0.12 vs. 0.30 ± 0.14		0.4604	0.47	−1.32 — 0.35	0.27 ± 0.12 vs. 0.28 ± 0.11		0.3048	0.54	−0.28 — 1.39
	Esc	0.20 ± 0.07 vs. 0.25 ± 0.15		0.7291	0.41	−0.46 — 1.31	0.21 ± 0.10 vs. 0.2 ± 0.09		>0.9999	0.16	−1.05 — 0.71
	***Ctrl vs. Esc***										
	NNB	0.36 ± 0.28 vs. 0.20 ± 0.07		0.0067**	**1.62**	−2.66 — −0.69	0.22 ± 0.12 vs. 0.21 ± 0.10		>0.9999	0.02	−0.86 — 0.82
	LNB	0.30 ± 0.14 vs. 0.25 ± 0.15		0.6872	0.34	−0.51 — 1.21	0.28 ± 0.11 vs. 0.20 ± 0.09		0.1659	0.78	−1.70 — 0.09
**(D) KYNA/KYN**		***Interaction***					***Interaction***				
	Drug exposure × Phenotype		(1, 39) = 0.64	0.4300				(1, 39) = 0.002	0.9675		
		***Main Effects***					***Main Effects***				
	Drug exposure		(1, 39) = 4.03	0.0517				(1, 39) = 2.71	0.1079		
	Phenotype		(1, 39) = 1.80	0.1879				(1, 39) = 0.15	0.7006		
		***Pairwise Comparisons***					***Pairwise Comparisons***				
	***NNB vs. LNB***										
	Ctrl	2.20 ± 0.55 vs. 2.29 ± 0.71		>0.9999	0.14	−0.68 — 0.96	2.65 ± 0.64 vs. 2.72 ± 0.53		>0.9999	0.12	−0.7 — 0.94
	Esc	1.74 ± 0.48 vs. 2.09 ± 0.35		0.3034	**0.81**	−0.09 — 1.75	2.38 ± 0.51 vs. 2.44 ± 0.47		>0.9999	0.11	−0.76 — 0.99
	***Ctrl vs. Esc***										
	NNB	2.23 ± 0.55 vs. 1.74 ± 0.48		0.1042	**0.86**	−1.77 — −0.004	2.65 ± 0.64 vs. 2.38 ± 0.51		0.5173	0.44	−1.30 — 0.34
	LNB	2.29 ± 0.71 vs. 2.09 ± 0.35		0.8046	−0.34	−1.21 — 0.51	2.72 ± 0.53 vs. 2.44 ± 0.47		0.4898	0.54	−1.43 — 0.32
**(E) QA/KYN**		***Interaction***					***Interaction***				
	Drug exposure × Phenotype		(1, 39) = 0.0008	0.9776				(1, 39) = 0.0016	0.9680		
		***Main Effects***					***Main Effects***				
	Drug exposure		(1, 39) = 1.38	0.2469				(1, 39) = 9.79	0.0033**		
	Phenotype		(1, 39) = 0.0008	0.9772				(1, 39) = 0.678	0.4153		
		***Pairwise Comparisons***					***Pairwise Comparisons***				
	***NNB vs. LNB***										
	Ctrl	6.19 ± 1.93 vs. 6.21 ± 1.25		>0.9999	0.02	−0.8 — 0.83	7.97 ± 1.65 vs. 8.38 ± 1.06		>0.9999	0.28	−0.53 — 1.11
	Esc	5.68 ± 1.14 vs. 5.68 ± 1.15		>0.9999	0.00	−0.88 — 0.88	6.30 ± 1.61 vs. 6.76 ± 2.38		>0.9999	0.21	−0.66 — 1.1
	***Ctrl vs. Esc***										
	NNB	6.19 ± 1.93 vs. 5.68 ± 1.14		0.8348	0.30	−1.15 — 0.54	7.97 ± 1.65 vs. 6.30 ± 1.61		0.0584	**0.98**	−1.91 — −0.12
	LNB	6.21 ± 1.25 vs. 5.68 ± 1.15		0.8089	0.42	−1.3 — 0.43	8.38 ± 1.06 vs. 6.76 ± 2.38		0.0736	**0.86**	−1.79 — 0.01
**(F) QA/KYNA**		***Interaction***					***Interaction***				
	Drug exposure × Phenotype		(1, 39) = 2.32	0.1358				(1, 39) = 0.0477	0.8283		
		***Main Effects***					***Main Effects***				
	Drug exposure		(1, 39) = 0.85	0.3637				(1, 39) = 2.79	0.1027		
	Phenotype		(1, 39) = 1.41	0.2416				(1, 39) = 0.11	0.7451		
		***Pairwise Comparisons***					***Pairwise Comparisons***				
	***NNB vs. LNB***										
	Ctrl	2.81 ± 0.65 vs. 2.91 ± 1.09		>0.9999	0.11	−0.71 — 0.93	3.20 ± 1.06 vs. 3.23 ± 0.93		>0.9999	0.03	−0.79 — 0.85
	Esc	3.54 ± 1.43 vs. 2.733 ± 0.444		0.1424	0.73	−1.66 — 0.16	2.68 ± 0.93 vs. 2.83 ± 0.97		>0.9999	0.18	−0.69 — 1.07
	***Ctrl vs. Esc***										
	NNB	2.81 ± 0.65 vs. 3.54 ± 1.43		0.1779	0.65	−0.20 — 1.53	3.20 ± 1.06 vs. 2.676 ± 0.934		0.3696	0.58	−1.46 — 0.26
	LNB	2.91 ± 1.09 vs. 2.73 ± 0.44		>0.9999	0.21	−1.07 −0.65	3.23 ± 0.93 vs. 2.83 ± 0.97		0.6308	0.41	−1.29 — 0.44

KYN/TRP, kynurenine/tryptophan; 5-HT/TRP, serotonin/tryptophan; 5-HIAA/5-HT, 5-hydroxyindoleacetic acid/serotonin; KYNA/KYN, kynurenic acid/kynurenine; QA/KYN; quinolinic acid/kynurenine; QA/KYNA, quinolinic acid/kynurenic acid; NNB, normal nest building; LNB, large nest building; PFC, prefrontal cortex; STR, striatum.

**Table 3.** Descriptive statistics of plasma LPS and LBP concentrations

		*LPS*					*LBP*				
		Mean ± SD (ng/ml)	*F*	*p*	*d*	CI*d*	Mean ± SD (ng/g)	*F*	*p*	*d*	CI*d*
**(A)**		***Interaction***					***Interaction***				
	Drug exposure × Phenotype		(1, 40) = 11.24	0.0018**				(1, 44) = 0.4157	0.5225		
		***Main Effects***					***Main Effects***				
	Drug exposure		(1, 40) = 6.507	0.0147*				(1, 44) = 0.01313	0.9093		
	Phenotype		(1, 40) = 5.287	0.0268*				(1, 44) = 9.667	0.0033*		
		***Pairwise Comparisons***					***Pairwise Comparisons***				
	***NNB vs. LNB***										
	Ctrl	1.69 ± 0.59 vs. 1.85 ± 0.56		0.9217	0.27	−0.57 — 1.12	549.39 ± 147.24 vs. 420.76 ± 133.4		0.1768	**0.88**	−1.75 — −0.06
	Esc	1.82 ± 0.51 vs. 0.96 ± 0.27		0.0005***	**1.99**	−3.11 — −1	577.06 ± 200.65 vs. 381.13 ± 225.9		0.022*	**0.89**	−1.75 — −0.06
	***Ctrl vs. Esc***										
	NNB	1.69 ± 0.59 vs. 1.82 ± 0.51		>0.9999	0.21	−0.58 — 1.02	549.39 ± 147.24 vs. 577.06 ± 200.65		>0.9999	0.15	−0.65 — 0.96
	LNB	1.85 ± 0.56 vs. 0.96 ± 0.27		0.0005***	**1.97**	−3.14 — −0.94	420.76 ± 133.4 vs. 381.13 ± 225.9		>0.9999	0.21	−1.01 — 0.59

LPS, lipopolysaccharide; LBP; lipopolysaccharide binding protein; NNB, normal nest building; LNB, large nest building.

## Supporting information

Karsten et al. supplementary material 1 Karsten et al. supplementary material

Karsten et al. supplementary material 2 Karsten et al. supplementary material

Karsten et al. supplementary material 3 Karsten et al. supplementary material

Karsten et al. supplementary material 4 Karsten et al. supplementary material

## Supplementary material

The supplementary material for this article can be found at https://doi.org/10.1017/neu.2025.16.
