# Supplementary figures and images for: Escitalopram alters tryptophan metabolism, plasma lipopolysaccharide, and the inferred functional potential of the gut microbiome in deer mice showing compulsive-like rigidity
Source: Acta Neuropsychiatr. 2025 Apr 3;37:e60. doi: 10.1017/neu.2025.16 (PMC13130255; doi:10.1017/neu.2025.16)

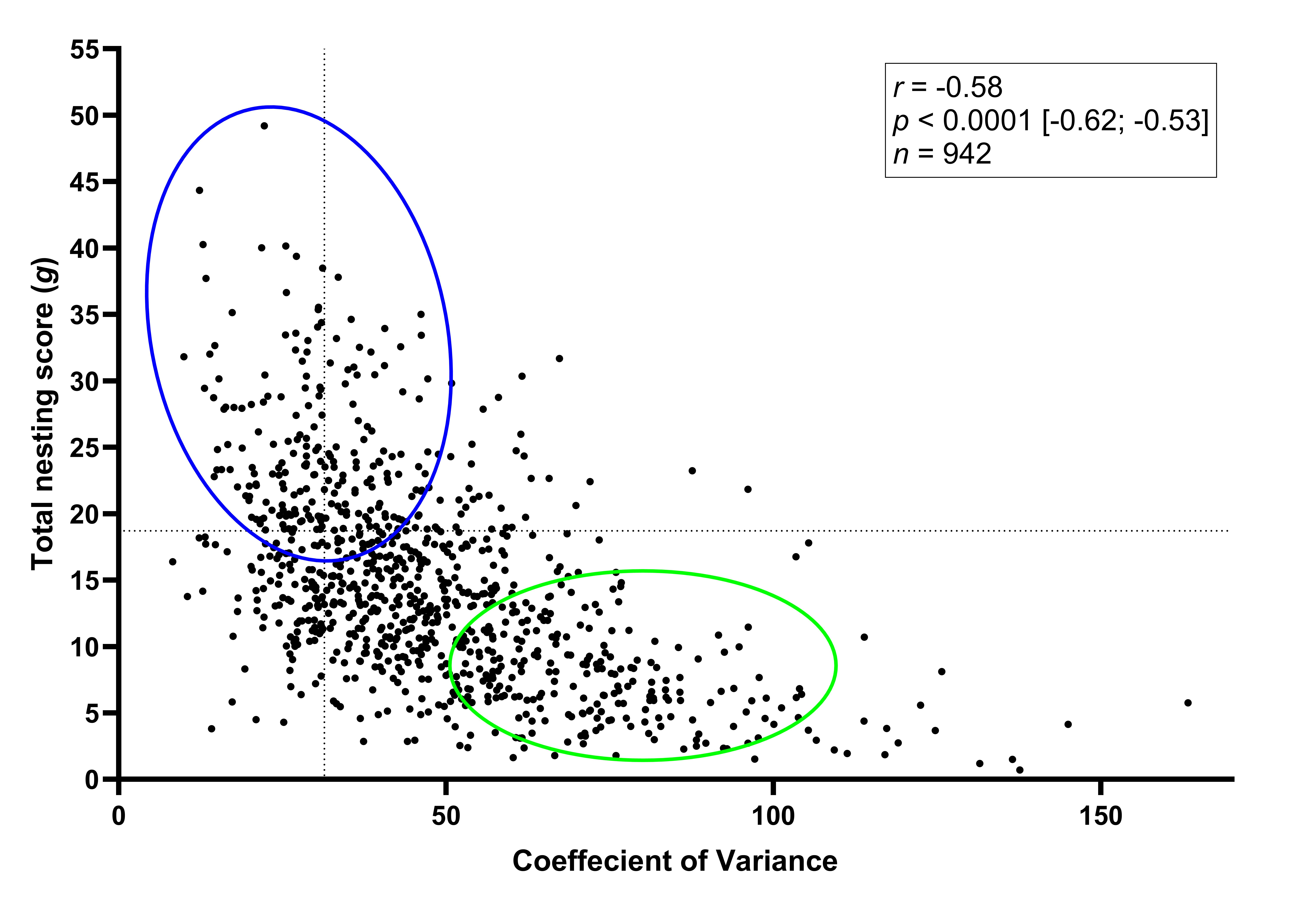

Supplement: Karsten et al. supplementary material 1 — Karsten et al. supplementary material [file S092427082500016Xsup001.zip › Fig. S1.jpg]

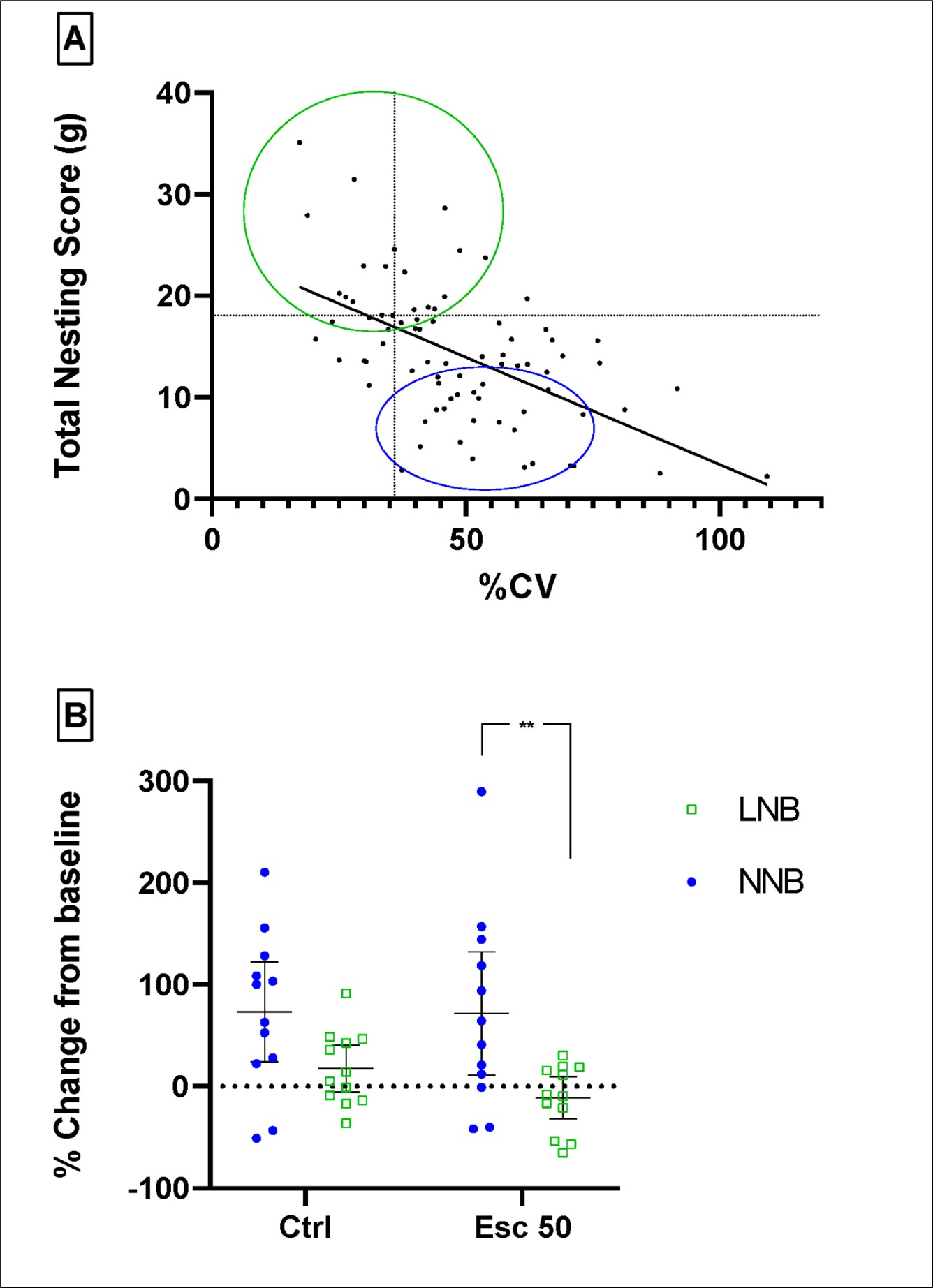

Supplement: Karsten et al. supplementary material 2 — Karsten et al. supplementary material [file S092427082500016Xsup002.zip › Fig. S2.jpg]

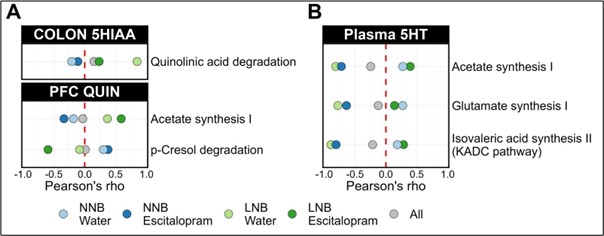

Supplement: Karsten et al. supplementary material 3 — Karsten et al. supplementary material [file S092427082500016Xsup003.zip › Fig. S3.jpg]
