# Supplementary material for: Escitalopram alters tryptophan metabolism, plasma lipopolysaccharide, and the inferred functional potential of the gut microbiome in deer mice showing compulsive-like rigidity
Source: Acta Neuropsychiatr. 2025 Apr 3;37:e60. doi: 10.1017/neu.2025.16 (PMC13130255; doi:10.1017/neu.2025.16)
Supplement: Karsten et al. supplementary material 4 — Karsten et al. supplementary material [file S092427082500016Xsup004.docx]

Supplementary Figure Captions

**Fig. S1:** **(A)** Spearman’s rank order correlation of nesting data (*n* = 942), collated from all prior studies in our lab, showing the bidirectional separation of normal (NNB) and large nesting (LNB) mice. Vertical dotted line: 25^th^ percentile of coefficient of variance scores; Horizontal dotted line: 75^th^ percentile of total nesting scores; data points enclosed in blue: mice selected for LNB; data points enclosed in green: mice selected for NNB.

**Fig. S2:** **(A)** Pre-selection nest-building data, and **(B)** percentage change in the total nesting scores of NNB- and LNB-expressing mice as a function of control or escitalopram intervention. Spearman’s rank order correlation of the total nesting scores (g) and the coefficients of variance with respect to the between-day nesting scores [r*_s_*(77) = -0.54, *p* < 0.0001]. Horizontal dotted line: 75^th^ percentile of total nesting scores generated; vertical dotted line: 25^th^ percentile with respect to the coefficients of variance; data of 79 animals are indicated. Blue circle: mice selected for LNB; green circle: mice selected for NNB. **(B)** Two-way ANOVA, ***p* = 0.0073. Data represented as mean ± 95% CI. Ctrl: control; Esc: escitalopram; NNB: normal nest building; LNB: large nest building.

**Fig. S3:** Correlations between host metabolites in different tissue and the inferred neuroactive potential are affected by escitalopram differentially, depending on the baseline nesting expression of the host. **(A)** Correlations between host metabolites and inferred gut-brain modules (GBMs) that are influenced by the baseline nesting expression of the host. **(B)** Correlations that depend on an interaction between the baseline nesting expression and escitalopram exposure. NNB mice exposed to water are depicted in light blue, NNB mice exposed to escitalopram are depicted in dark blue, LNB mice exposed to water are depicted in light green, and LNB mice exposed to escitalopram are depicted in dark green. NNB: normal nest building; LNB: large nest building. The average Pearson’s correlations along groups are represented as grey dots labelled as “All”.
